# Inequality of opportunity in healthcare expenditures: evidence from China

**DOI:** 10.1186/s12913-020-05252-z

**Published:** 2020-05-06

**Authors:** Yuyang Zhang, Peter C. Coyte

**Affiliations:** 1 School of Public Economics and Administration, Shanghai University of Finance and Economics, 777 Guoding Road, Shanghai, 200433 China; 2 Institute of Health Policy, Management and Evaluation, University of Toronto, 155 College Street, Toronto, ON M5T 3M6 Canada

**Keywords:** Inequality of opportunity, Circumstance-free effort, Outpatient care, Inpatient care

## Abstract

**Background:**

The theory of equality of opportunity attributes total inequality to effort levels and circumstance factors. Inequality attributable to circumstance is defined as inequality of opportunity (IOp), namely inequity. Many studies have been pursued in this area but few concerning health care, especially in China. Despite Chinese health system reforms, healthcare inequity remains. This study explores the extent and sources of IOp in outpatient and inpatient expenditures in China.

**Methods:**

We used three waves (2011, 2013 and 2015) of data from the China Health and Retirement Longitudinal Study that offer a nationally representative sample of Chinese residents aged 45 and older. Based on a pooled regression model, we estimated the contribution of circumstance factors to the inequality in outpatient and inpatient expenditures by defining a counterfactual distribution. The “circumstance-free effort” was introduced to deal with the correlation between circumstance and effort.

**Results:**

We report a decline in inequity from 2011 to 2015, and the IOp ratio to total inequality in outpatient and inpatient expenditures decreased 9.4% (from 28.6 to 25.9%) and 3.3% (from 49.1 to 47.5%), respectively. Social background, medical supply-side factors, including the type of basic medical insurance, region and community medical resources were important sources of IOp in outpatient and inpatient expenditures.

**Conclusions:**

These findings provide information on which to base policies designed to reduce inequity in healthcare expenditures. It is necessary to transfer more subsidies to the New Co-operative Medical System, and to address the uneven regional distribution of medical resources. Additionally, increasing access to quality primary community clinics may be a pro-poor policy to alleviate inequity in the use of outpatient care. Compared to outpatient services, policies protecting vulnerable populations need to pay more attention to the financing and design of inpatient services.

## Background

The theory of equality of opportunity proposed by Roemer [1–3] is associated with the responsibility principle and offers an appropriate interpretive framework in which to define equity. It attributes total inequality to levels of *effort*, which individuals ought to be held responsible for, and *circumstance* factors, which are outside the sphere of individual responsibility. Inequality attributable to circumstance factors is here referred to as inequality of opportunity (i.e. IOp), namely inequity. This framework prompted empirical studies in a range of fields, primarily with respect to income [3–6], education [7–10], and more recently, health [11–15].

The concept of IOp was already implicit in much of the empirical research on health economics [16], before Fleurbaey and Schokkaert [17, 18] introduced it to health and healthcare. For example, healthcare inequality caused by need is usually considered to be fair (effort), while many studies have focused on healthcare inequality attributable to socioeconomic status (i.e. SES), with such inequality deemed unfair (circumstance). These studies [19, 20] analyzed a “partial” inequity only associated with the SES factor, while the IOp framework could include any other relevant factors, such as medical supply [17].

It is very important to study the IOp in healthcare, especially in China, where the healthcare system is facing a range of challenges due to its resource poor status. Reforms to China’s public health system have been prioritized since the start of the twenty-first century. In order to promote equity in the economic accessibility of healthcare for all residents, three separate medical insurance plans were launched: the Urban Employee Basic Medical Insurance (UEBMI) scheme, designed for employed urban residents; the Urban Resident Basic Medical Insurance (URBMI) scheme, designed for non-employed urban residents; and the New Co-operative Medical System (NCMS), designed for the rural population. According to the *National Healthcare Security Administration*, by the end of 2018, more than 95% of the Chinese population had basic medical insurance coverage. Despite almost universal coverage, there are still wide variations in the level of finance, method of organization and benefits covered by each insurance scheme. The UEBMI is jointly funded by employers and employees, with annual funding set to be at least 8% of employees’ annual wages. The URMBI and NCMS were designed to provide insurance for residents without stable jobs and income, and are funded through both government subsidies and individual premiums. In 2018, per-capita expenditures under the UEBMI were 3316.68 *yuan*, while equivalent figures for the URMBI and the NCMS were only 700.29 *yuan* and 627.57 *yuan,* respectively. Such funding variations account for the wide differences in benefits between insurance schemes. Moreover, the funding gap is not only reflected in the reimbursement ratio, but also in the service package. Compared with comprehensive coverage under the UEBMI, the other two insurance plans’ have focused coverage to inpatient care and catastrophic illness insurance for outpatient services; some basic outpatient services are not insured [21].

While there have been improvements in equity due to health insurance reforms in China [22, 23], inequity in healthcare remains. Different from other countries, the household registration system in China, which identifies a person as a rural or urban resident, is an important classification basis of the above medical insurance plans. The fact that the availability of each type of medical insurance scheme depends entirely on one’s social status [24], will itself result in inequity. It means that, the inequality of opportunity in healthcare associated with the characteristics of the healthcare system in China deserves more attention.

There are to date a paucity of empirical applications on healthcare in China, and only Sun et al. [25] explored the inequity of individual healthcare expenditures based on the theory of equality of opportunity, without distinguishing outpatient and inpatient services. As there may exist heterogeneity in the inequity in outpatient and inpatient care [22, 26, 27], it would be useful to analyze each component of care separately. The objective of this study was to estimate the extent and sources of inequity in two separate types of medical expenditures (i.e., outpatient and inpatient expenditures) among Chinese residents, based on the framework of equality of opportunity. We designed two types of circumstance variables, i.e., socioeconomic status and medical supply-side factors (including the type of basic medical insurance, region and community medical resources), to explore the sources of IOp. This knowledge may be gleaned to inform policymakers on ways to design policies to reduce inequity in healthcare expenditures and optimize the use of medical resources.

## Methods

### Design of the study

This study measured the IOp in healthcare based on two approaches, i.e., *direct unfairness* and *fairness gap*, proposed by Fleurbaey and Schokkaert [17, 18], which should satisfy two conditions correspondingly:

**Condition****1** (*reward principle*): A measure of unfair inequality should not reflect legitimate variation in outcomes. This principle rewards effort among individuals with identical circumstances.

**Condition****2** (*compensation principle*): If a measure of unfair inequality is zero, there should be no illegitimate differences left, i.e. two individuals with the same effort should have the same outcome.

The *direct unfairness* satisfies the reward principle. It removes legitimate sources of variation by fixing effort of individual *i*, *E*_*i*_, with a series of reference values, $\tilde{E}$, in order to achieve outcome variations that are due exclusively to circumstances (*C*_*i*_,) of individual *i*, i.e. ${\tilde{y}}_i=f\left({C}_i,\tilde{E}\right)$. Inequality in the distribution of ${\tilde{y}}_i$, which refers to the IOp, can immediately be measured with an inequality index *I*(•). The *fairness gap* satisfies the compensation principle. It first defines a fair counterfactual distribution without illegitimate inequality, i.e. ${y}_i^{\ast }=f\left({C}^{\ast },{E}_i\right)$, with *C*_*i*_ fixed at C^*^. The IOp can be measured as *I*(Δ_*i*_), where ${\Delta}_i={y}_i-{y}_i^{\ast }$ (absolute measure) or ${\Delta}_i={y}_i/{y}_i^{\ast }$ (relative measure).

It should be noted that the reward and compensation principles are only compatible under one situation that *C*_*i*_ and *E*_*i*_ are completely independent [17, 18]. However, the correlation between *C*_*i*_ and *E*_*i*_ is an essential consideration [17], because effort may be determined by factors outside individual responsibility [2]. In order to keep *C*_*i*_ and *E*_*i*_ separable, we followed the approach proposed by Jusot et al. [13], i.e., effort may be purged of any contamination coming from circumstances by estimating an auxiliary equation. We assumed that this function is linear:

1 $$ {E}_i=\eta +\lambda {C}_i+{e}_i $$

The residual term *e*_*i*_, represents the purged efforts after removing the circumstance effect. An equation accounting for variations in individual expenditures on healthcare, denoted by *hc*_*i*_, can be written in log-linear form as follows, after controlling for demographics, *D*_*i*_:

2 $$ \ln \left({hc}_i\right)=\alpha +\beta {C}_i+\gamma {\hat{e}}_i+\varphi {D}_i+{u}_i $$

where ${\hat{e}}_i$ denotes the estimated value of the residual term *e*_*i*_ in (1), which can be interpreted as *circumstance-free effort*; *u*_*i*_ represents unobservable factors. So, the function of *hc*_*i*_ is multiplicatively separable:

3 $$ {hc}_i=\exp \left(\alpha +\varphi {D}_i+{u}_i\right)\cdot \exp \left(\beta {C}_i\right)\cdot \exp \left(\gamma {\hat{e}}_i\right) $$

Then we employed a Gini Coefficient to the inequality index *I*(•) to calculate the value of IOp. Both the direct unfairness and fairness gap approaches yielded the same IOp, regardless of the reference values selected, since the Gini Coefficient is scale-invariant. Based on Eq. (3), we can get the IOp of healthcare expenditures as follows:

4 $$ IOp= Gini\left(h{\tilde{c}}_i\right)= Gini\left(\exp \left(\alpha +\varphi \tilde{D}+{u}_i\right)\cdot \exp \left(\beta {C}_i\right)\cdot \exp \left(\gamma \tilde{\hat{e}}\right)\right)\kern0.5em = Gini\left({\Delta}_i\right)= Gini\left({hc}_i/{hc}_i^{\ast}\right)= Gini\left(\exp \left(\beta {C}_i\right)/\exp \left(\beta {C}^{\ast}\right)\right) $$

Where $\tilde{\hat{e}}$, $\tilde{D}$ and C^*^ denote the reference values of ${\hat{e}}_i$, *D*_*i*_ and *C*_*i*_, respectively.

Following Rosa Dias [11] and Juarez and Soloaga [28], we further calculated the ratio of IOp to total inequality by dividing the IOp by the same metric *I* (•) applied to the actual distribution of healthcare expenditures. Then the IOp ratio can be expressed as *IOp*/*Gini*(*hc*_*i*_). It can be noted that, compared with the traditional regression approach, the IOp model allowed us to further calculate the total contribution of circumstance variables to the variations of outcomes.

### Data

Data in this study were derived from three waves (2011, 2013 and 2015) of the China Health and Retirement Longitudinal Study (CHARLS), which was conducted by the China Centre for Economic Research of Peking University. The sample is representative of community dwelling Chinese residents aged 45 and over. The design of CHARLS questionnaire was based on the Health and Retirement Study (HRS) and related aging surveys, such as the English Longitudinal Study of Aging (ELSA) and the Survey of Health, Aging and Retirement in Europe (SHARE). Through face-to-face interview, CHARLS collected data from respondents, including demographics, socioeconomic status, health and healthcare information (such as health status, health-related behaviors, health insurance and health services use) and community level information. The sampling was carried out at four levels: the county and neighborhood levels employed the PPS (probabilities proportional to size) sampling, while the household and individual levels employed random sampling. All the questionnaires and data are open to researchers all over the world and could be downloaded at the official website (charls.pku.edu.cn).

The 2011 national baseline survey was conducted in 28 provinces, 150 countries/districts, 450 villages/urban communities, across the country, including about 10,000 households and 17,500 individuals with an average response rate of 80.5% [29]. Two follow-up surveys were conducted in 2013 and 2015. In the database of CHARLS in three waves, there were a total of 49,265 respondents aged 45 and over. We selected 5472 respondents who had received outpatient care during the past 1 month or inpatient care during the past 1 year. After excluding the observations where we lacked information for effort or circumstance variables, our analyses were performed on an unbalanced set of data with 5079 observations.

### Variables

We followed the model proposed by Fleurbaey and Schokkaert [18], which specified individual healthcare to be a function of medical needs, social background, individual preferences, supply-side factors and available information. Compared with the classic model of healthcare use advanced by Anderson and Newman [30] and Anderson [31], the Fleurbaey and Schokkaert model is designed to identify the distinguishing role played by effort and circumstance in accounting for variations in healthcare use. It is therefore a simplified model, but it covers most of the factors associating with healthcare use included in more general models, such as the Anderson and Newman model.

The key to applying the theory of equality of opportunity is to determine the boundary between effort and circumstance. In healthcare applications, the effort variables are defined as those that lead to legitimate inequality in healthcare, while inequality due to circumstance factors are illegitimate [17]. There seems to be consensus that differences in healthcare expenditures caused by need variables are legitimate, but there is some debate about individual preferences. Rawls [32, 33] and Dworkin [34–36] suggested that individuals should be held responsible for their preferences. Some authors objected to this view and argued that preferences are often the product of circumstances [2, 37, 38]. We followed the view of the US Institute of Medicine [39], which suggested that medical needs and preferences were both legitimate sources of healthcare differences. We defined socioeconomic status (SES), which lead to illegitimate inequality, as circumstance factors [17, 40]. Individuals cannot be held responsible for medical supply-side influences, so supply variables are also illegitimate sources of inequality [18], i.e. circumstance factors.

Table 1 shows the list of variables used for IOp estimation and the associated definitions.

**Table 1:** Variable Definitions

Variables	Definition and Description
Outcome variables	
Expenditures of Outpatient care	Total medical cost of outpatient care during the past 1 month (yuan)
Expenditures of Inpatient care	Total medical cost of inpatient care during the past 1 year (yuan)
Effort Variables	
Self-assessed health	1 = Good, 0 = Not Good
Chronic disease	1 = Yes, 0 = No
Physical examination	1 = Yes, 0 = No
Supplementary insurance	1 = Yes, 0 = No
Circumstance variables	
Job	1 = Agricultural work, 0 = Others
Income	Household Income Per Capita (thousand yuan/year)
Income^2^	Quadratic income
Educational attainment	1 = Educated, 0 = No formal education
Type of basic medical insurance	1 = New Co-operative Medical System (NCMS), 0 = Other basic medical insurances
Region	1 = East provinces; 0 = Others
Primary medical clinic	1 = Yes;0 = No
Hospital	1 = Yes; 0 = No
Demographics	
Gender	1 = Male; 0 = Female
Age	≥45 years

#### Healthcare expenditures

Medical expenditures were stratified into two components to assess whether inequality of opportunity varied across those components: outpatient expenditures; and inpatient expenditures. Respondents were asked about the total cost (including out-of-pocket and the part reimbursed by medical insurance) of all outpatient visits during the past 1 month and inpatient care during the past 1 year. The outpatient and inpatient expenditures were both expressed in 2015 Yuan prices using consumer price indices specific to each wave.

#### Effort variables

We used two dummies indicating *self-assessed health* (SAH) and *the presence of chronic diseases* to capture individual needs for healthcare [19]. SAH was derived from responses to the question in CHARLS, ‘Would you say your health is very good, good, fair, poor or very poor?’. We grouped very good and good health (1 = Good) against fair, poor and very poor health (0 = Not good) in line with previous literature so as to simplify the empirical analysis [13]. The presence of at least one chronic condition was adopted as the second indicator of healthcare need. Respondents were asked if they had been diagnosed with any of the 14 chronic conditions listed, including hypertension, dyslipidemia, diabetes or high blood sugar and so on.

We designed two proxy variables to represent preferences. One was based on the question that asked respondents whether they had participated in *supplementary medical insurance*. The other question concerned the use of a *physical examination* in the past 2 years. Individuals who purchase supplementary insurance or receive a physical examination are viewed as having a higher preference for medical expenditures.

#### Circumstance

Individuals’ SES were measured by *Job*, *household income* per capita and *educational attainment*. Job was designed as a dummy capturing whether one was engaged in agricultural work. Household income per capita was expressed in 2015 Yuan prices and measured in thousands of yuan. We also introduced a quadratic term for the income variable. Educational attainment was measured by a dummy variable that captured whether respondents received formal education. Before the 1960s, educational resources were scarce in China. Approximately 30% respondents had no formal education and almost 90% only had a middle school education or less.

We used the availability of medical services and the characteristics of the healthcare system to represent supply-side influences. The availability of healthcare services was described by *region* (i.e. the Eastern provinces or others) and two dummies extracted from the community questionnaire: whether there were *primary medical clinics* or *hospitals* in the community/village. The characteristics of the healthcare system, depends largely on healthcare policies. The *type of basic medical insurance* may be an appropriate proxy variable for policy. Considering the NCMS is specially targeted to the rural population and more than 75% of the sample were covered under the NCMS plan, we defined this policy variable as a dummy variable with ‘1 = NCMS, 0 = others’.

#### Demographics

In addition to circumstance and effort factors, the analysis controlled for the demographic characteristics: *gender* and *age*. Since demographics sometimes were taken as biological indicators of individual needs for medical care [19], we put them into the basket of effort variables when IOp was measured [13].

### Data analysis

We estimated the determinants of medical expenditures (Eq. (2)) using a pooled regression model with all waves of the CHARLS. Clustered standard errors were employed at the individual level, to allow for the correlation of identical individual’s residuals in different waves [41]. It was not feasible to implement a fixed effects specification, because some circumstance variables that did not change with time (i.e., education, region and community variables) would be omitted. A random effects model imposes stronger exogeneity assumptions and its estimates were very similar to those obtained from the pooled model [42]. We therefore employed the pooled model. Additionally, because medical expenditures were observed only for service users, the potential for sample selection bias exists. We used the Heckman selection correction model to estimate the Inverse Mills Index using ‘have received outpatient (inpatient) care or not’ as the dependent variable of the selection function. We found that the null hypothesis of the absence of selection bias was not rejected which meant that our model did not have selection bias.

It should be noted that we used several explanatory variables that might be correlated to each other (such as job and type of basic medical insurance). We therefore checked for multicollinearity effects by calculating the Variance Inflation Factors (VIF). The VIF values for all explanatory variables ranged from 1.01 to 3.64 (below the critical value of 5), which indicated no collinearities could be seen in the regression model.

## Results

**Fig. 1:**
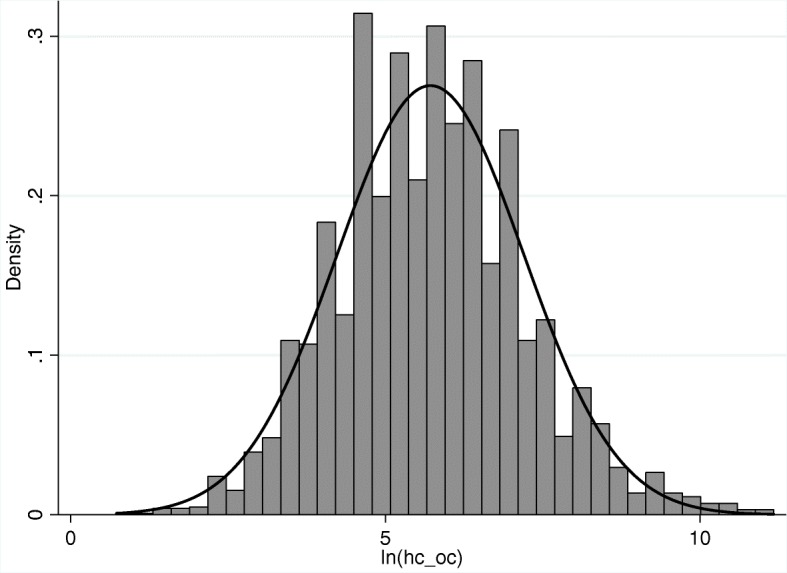
Distribution of logarithmic outpatient expenditures

**Fig. 2:**
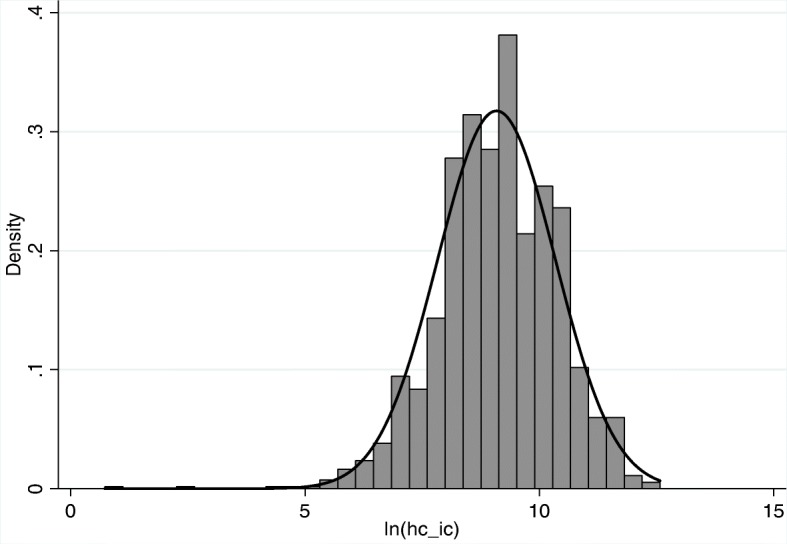
Distribution of logarithmic inpatient expenditures

Given each expenditure variable was right-skewed, these variables were logarithmically transformed to address potential concerns of heteroscedasticity. After transformation, these variables were approximately normally distributed, see Figs. 1 and 2. Table 2 further compares the descriptive statistics of variables in 2011, 2013 and 2015.

**Table 2:** Descriptive Statistics of Variables

Variables	Mean			SD		
	2011	2013	2015	2011	2013	2015
Outpatient care expenditures	725.5	1059.96	1415.71	2395.80	3658.44	4217.65
Inpatient care expenditures	14,782.92	17,139.52	17,395.42	21,223.60	24,570.68	22,093.50
SAH: Good	0.08	0.08	0.08	0.27	0.27	0.28
Chronic disease	0.86	0.80	0.77	0.35	0.40	0.42
Physical examination	0.56	0.53	0.50	0.50	0.50	0.50
Supplementary insurance	0.03	0.09	0.05	0.17	0.29	0.23
Job: Agricultural work	0.50	0.50	0.49	0.50	0.50	0.50
Income	13.42	12.37	8.64	23.89	26.33	20.83
Income^2^	750.56	845.91	508.37	8140.06	18,369.47	5870.17
Educational attainment: Educated	0.68	0.71	0.76	0.47	0.45	0.42
Type of basic medical insurance: NCMS	0.77	0.77	0.76	0.42	0.42	0.43
Region: East provinces	0.30	0.27	0.27	0.46	0.44	0.44
Primary medical clinic	0.78	0.78	0.78	0.42	0.42	0.42
Hospital	0.09	0.09	0.09	0.29	0.29	0.28
Gender: Male	0.42	0.43	0.43	0.49	0.50	0.50
Age	61.53	61.57	60.87	10.05	9.72	9.67
Obs.	1585	1940	1554	1585	1940	1554

Table 3 shows the full sample regressions for expenditures on outpatient and inpatient care. There were four main sets of results. First, all the effort variables (including SAH, chronic disease, physical examination and supplementary insurance) were significantly associated with outpatient expenditures (i.e., individuals with higher medical needs and stronger preferences had larger outpatient expenditures), while having a physical examination was the sole effort variable (positively) related to the inpatient expenditures. Second, all SES variables were strongly associated with both types of medical expenditures. Agricultural workers consumed less outpatient and inpatient services. There was an inverted U-shaped relationship between each medical expenditure category and income. The value of income corresponding to the peak of the parabola showed that except for a very small number of individuals (less than 1%), most of analysis sample was distributed on the rising segment of the function. Educated individuals consumed more outpatient services, but there was no significant effect on inpatient care expenditures. Third, for supply variables, non-NCMS participants and Eastern residents consumed more outpatient and inpatient services than their counterparts. While community medical resources had no effect on inpatient expenditures, individuals living in a community with a hospital had larger outpatient expenditures. Unusually, the availability of a primary medical clinic in the community was negatively associated with individuals’ outpatient expenditures. Fourth, for demographic variables, males had larger inpatient expenditures, and both outpatient and inpatient expenditures tended to fall with age.

**Table 3:** Pooled regression estimates on outpatient and inpatient expenditures

Variables	Outpatient care expenditures: ln (*hc_oc*)		Inpatient care expenditures: ln (*hc_ic*)	
Good SAH	− 0.075^***^	(0.024)	− 0.022	(0.033)
Chronic disease	0.056^**^	(0.023)	−0.031	(0.036)
Physical examination	0.143^***^	(0.022)	0.068^**^	(0.031)
Supplementary insurance	0.040^*^	(0.023)	0.028	(0.031)
Agricultural work	−0.417^***^	(0.052)	−0.538^***^	(0.073)
Income	0.004^**^	(0.002)	0.010^***^	(0.003)
Income^2^	−3.45e-06^*^	(0.000)	−3.79e-05^***^	(0.000)
Educated	0.197^***^	(0.057)	0.021	(0.080)
NCMS	−0.261^***^	(0.063)	− 0.402^***^	(0.083)
East region	0.110^**^	(0.054)	0.433^***^	(0.077)
Primary medical clinic	−0.120^**^	(0.058)	−0.017	(0.073)
Hospital	0.233^***^	(0.084)	0.117	(0.100)
Male	0.003	(0.050)	0.251^***^	(0.071)
Age	−0.006^**^	(0.003)	−0.014^***^	(0.003)
Constant	6.371^***^	(0.197)	10.071^***^	(0.265)
Obs.	4070		1323	

Note: Standard errors are in parenthesis; ^***^p < 0.01, ^**^p < 0.05, ^*^p < 0.1

Table 4 shows the measurement of IOp in the full sample and how it changed over each wave. For all waves, the inequality of opportunity in inpatient expenditures (0.274) was greater than that for outpatient expenditures (0.207), while total inequality was in the opposite direction. About 27.3 and 47.7% of total inequality in outpatient and inpatient expenditures was attributed to inequality of opportunity, respectively. The IOp for each category of medical expenditures fell over the study period from 2011 to 2015. While total inequality in outpatient expenditures increased over the study period, total inequality in inpatient expenditures peaked in 2013 but fell to its lowest level in 2015. Due to the increase in total inequality in outpatient expenditures, its IOp ratio further decreased from 28.6% in 2011 to 25.9% in 2015. With the change of total inequality, the IOp ratio in inpatient expenditures declined from 49.1 to 46.1% and then rebounded to 47.5% in 2015.

**Table 4:** Measurement of inequality of opportunity

	Outpatient care expenditures: *hc_oc*				Inpatient care expenditures: *hc_ic*			
	2011	2013	2015	All waves	2011	2013	2015	All waves
IOp	0.210	0.210	0.200	0.207	0.283	0.272	0.263	0.274
Total inequality	0.733	0.743	0.772	0.757	0.576	0.590	0.554	0.575
IOp Ratio	0.286	0.283	0.259	0.273	0.491	0.461	0.475	0.477
Obs.	1320	1542	1208	4070	338	521	464	1323

Table 5 further illustrates the comparison of regression results between different groups classified by their demographic characteristics. The corresponding IOp measurement were in Table 6. We found that group heterogeneity of IOp was more obvious in inpatient expenditures than in outpatient expenditures, and the variation between different age groups was greater than that between men and women. For women, the IOp ratio in outpatient expenditures (27.5%) was slightly higher than that for men (27.2%), but in the case of inpatient expenditures it was much lower (44.9%) than that for men (52.2%). The elderly had a lager IOp value (0.232) but a smaller IOp ratio (26.8%) for outpatient expenditures, than individuals under 60 years of age (0.196 and 28.1% for IOp value and IOp ratio, respectively). But in the case of inpatient expenditures, the IOp value (0.301) and its ratio (53.2%) for the elderly were both lager than those for younger individuals (0.248 and 42.3%, respectively), as total inequality for the elderly was smaller.

**Table 5:** Regression estimates on outpatient and inpatient expenditures for subsamples comparison

	Outpatient care expenditures: ln (*hc_oc*)				Inpatient care expenditures: ln (*hc_ic*)			
	Male	Female	Age < 60	Age ≥ 60	Male	Female	Age < 60	Age ≥ 60
Good SAH	−0.083^**^	− 0.068^**^	− 0.110^***^	− 0.039	− 0.052	0.014	0.080	− 0.055
	(0.039)	(0.031)	(0.031)	(0.037)	(0.038)	(0.054)	(0.058)	(0.039)
Chronic disease	0.087^**^	0.036	0.058^*^	0.046	−0.074	0.003	− 0.051	− 0.016
	(0.036)	(0.030)	(0.031)	(0.035)	(0.050)	(0.051)	(0.057)	(0.046)
Physical examination	0.197^***^	0.106^***^	0.137^***^	0.141^***^	0.083^**^	0.048	0.058	0.083^**^
	(0.037)	(0.028)	(0.033)	(0.031)	(0.042)	(0.047)	(0.055)	(0.039)
Supplementary insurance	0.039	0.043	0.009	0.071^**^	0.037	0.022	0.104	−0.002
	(0.035)	(0.029)	(0.030)	(0.034)	(0.038)	(0.053)	(0.064)	(0.033)
Agricultural work	−0.427^***^	−0.410^***^	− 0.402^***^	−0.442^***^	− 0.505^***^	−0.588^***^	− 0.646^***^	−0.513^***^
	(0.083)	(0.066)	(0.076)	(0.072)	(0.099)	(0.107)	(0.120)	(0.092)
Income	0.004	0.004^**^	0.007^**^	0.005^**^	0.018^***^	0.009^**^	−0.004	0.013^***^
	(0.004)	(0.002)	(0.003)	(0.002)	(0.005)	(0.004)	(0.005)	(0.004)
Income^2^	−9.02e-06	−3.44e-06	−2.75e-05^**^	−4.50e-06^*^	−1.18e-05^***^	−3.10e-05^**^	4.05e-05	−5.25e-05^***^
	(0.000)	(0.000)	(0.000)	(0.000)	(0.000)	(0.000)	(0.000)	(0.000)
Educated	0.154	0.216^***^	0.351^***^	0.087	0.184	−0.079	−0.125	0.075
	(0.112)	(0.066)	(0.085)	(0.075)	(0.121)	(0.108)	(0.137)	(0.099)
NCMS	−0.345^***^	−0.183^**^	− 0.113	−0.392^***^	− 0.440^***^	−0.319^**^	− 0.382^***^	−0.394^***^
	(0.097)	(0.083)	(0.090)	(0.089)	(0.108)	(0.133)	(0.143)	(0.103)
East	0.116	0.108	0.001	0.193^**^	0.538^***^	0.336^***^	0.322^**^	0.480^***^
	(0.086)	(0.069)	(0.075)	(0.077)	(0.095)	(0.125)	(0.136)	(0.092)
Primary medical clinic	−0.043	−0.179^**^	−0.195^**^	−0.053	0.041	−0.077	−0.104	0.039
	(0.092)	(0.074)	(0.084)	(0.078)	(0.094)	(0.111)	(0.127)	(0.088)
Hospital	0.175	0.259^**^	0.249^**^	0.193	0.076	0.163	−0.289	0.261^**^
	(0.136)	(0.106)	(0.119)	(0.120)	(0.122)	(0.163)	(0.191)	(0.117)
Male	–	–	−0.020	0.031	–	–	0.242^**^	0.262^***^
	–	–	(0.072)	(0.070)	–	–	(0.118)	(0.090)
Age	−0.006	−0.006^*^	0.003	−0.011^**^	−0.008^*^	− 0.020^***^	−0.013	− 0.010^*^
	(0.004)	(0.003)	(0.008)	(0.005)	(0.005)	(0.005)	(0.014)	(0.006)
Constant	6.431^***^	6.349^***^	5.702^***^	6.790^***^	9.724^***^	10.548^***^	10.449^***^	9.674^***^
	(0.343)	(0.253)	(0.467)	(0.388)	(0.377)	(0.402)	(0.771)	(0.471)
Obs.	1657	2413	1948	2122	668	655	464	859

Note: Standard errors are in parenthesis; ^***^p < 0.01, ^**^p < 0.05, ^*^p < 0.1

**Table 6:** Measurement of inequality of opportunity for subsamples comparison

	Outpatient care expenditures: *hc_oc*				Inpatient care expenditures: *hc_ic*			
	Male	Female	Age < 60	Age ≥ 60	Male	Female	Age < 60	Age ≥ 60
IOp	0.207	0.207	0.196	0.232	0.297	0.260	0.248	0.301
Total inequality	0.761	0.753	0.734	0.775	0.569	0.579	0.586	0.566
IOp Ratio	0.272	0.275	0.281	0.268	0.522	0.449	0.423	0.532
Obs.	1657	2413	1948	2122	668	655	464	859

## Discussion

### Main findings

Several salient findings were revealed. First, despite observed improvement in healthcare equity over the study period, SES and medical supply-side factors were still important sources of the inequality of opportunity in outpatient and inpatient expenditures. Second, compared with outpatient expenditures, inpatient expenditures were more inequitable. Third, the level of inequity in outpatient expenditures was higher for women and younger individuals than for their counterparts, but the opposite relationship was found for inpatient expenditures.

While we found a downward trend in inequity in China from 2011 to 2015, inequity was still a serious problem, especially in the case of inpatient expenditures. The improvement shown in healthcare equity over our study period was consistent with the findings of Zhou et al. [22] and Zhou et al. [43]. Although together these results suggest that health reforms in China have been successful in yielding improvements in health inequity, inequality of opportunity, in our study, still accounted for a large share of total inequality (27.3% for outpatient care and 47.7% for inpatient care).

We noted that more serious inequity existed in inpatient care than in outpatient care. This gap may be because it is more difficult for individuals who are worse-off in terms of circumstance (e.g. the poor) to afford inpatient care than outpatient care. To be specific, outpatient services generally address common ailments. In China, the demand for outpatient services can often be met by both primary healthcare clinics and hospitals. It is therefore easier to achieve equity in outpatient care. But inpatient services have higher prices and require more medical resources and technical support. The magnitude of inpatient expenditures further worsens the situation for vulnerable groups. According to data from China’s *National Health Commission*, more than 40% of the poor households in China were in poverty due to illness in 2018. Inequity in inpatient expenditures therefore deserves greater attention by policy makers.

We confirmed previous studies that socioeconomic characteristics were important factors related to healthcare inequity in China [44]. Individuals who worked in agriculture and had lower incomes were at a disadvantage in both outpatient and inpatient expenditures. A positive association was also found between educational attainment and outpatient expenditures, but this relationship did not hold for inpatient expenditures. The finding of a differential effect on inpatient and outpatient expenditures diverged from some international literature, e.g. Channon et al. [45], which found that educational attainment was positively associated with longer hospital stays in Brazil. Working in agriculture, lack of education and having lower incomes are almost all concentrated among rural Chinese residents. To help rural residents reduce financial barriers to utilization, the Chinese government launched the NCMS in the early 2000s [21]. We found that the residents covered by the NCMS consumed less outpatient and inpatient services than those covered by the other basic medical plans. Previous literature has shown that the implementation of the NCMS increased health service utilization [46], but the effect was limited [47]. Our findings further confirmed the limited effect of the NCMS on both outpatient and inpatient expenditures. The low fund level for the NCMS relative to the other medical schemes [48] may lead to a larger gap in medical expenditures between rural residents, who are already at a socioeconomic disadvantage, and urban residents.

We found that the regional allocation of medical resources was also a key circumstance factor. Some studies [27] even suggested that region of residence was more important than individual characteristics in healthcare inequality. Our study showed that residents living in Eastern provinces consumed more outpatient and inpatient services, than the ones in the Central and Western provinces. The economic development in China is not balanced geographically, thus inequality in the geographic distribution of health resources is also evident [49]. We further found that the availability of hospitals or primary clinics in the community also led to differences in outpatient expenditures. Individuals living in a community with hospitals had larger outpatient expenditures, while the opposite relationship was found for the availability of primary medical clinics. These results implied that, the presence of a primary medical clinic in the community may act as a substitute for hospital outpatient care when patients have an opportunity to choose their healthcare setting. Table 2 showed that only 78% of respondents lived in communities with primary clinics. So, increasing access to primary clinics to all communities may help avoid the overuse of hospitals. Some studies have also suggested that Chinese patients tend to place limited trust in primary healthcare clinics due to their shortage of medical resources [49], leading to a tendency for patients to seek care from hospitals [50]. More resources need to be transferred to primary medical clinics to improve the perceived quality of their medical services, so as to increase public trust. This policy would also be useful to improve equity in the use of outpatient care by ensuring that patients seek medical care based on their needs, rather than their ability to pay.

Age- and sex-group heterogeneity of IOp was more obvious in inpatient expenditures than in outpatient expenditures. This finding suggests that policymakers focus on the characteristics of different groups when designing policies to improve equity of inpatient care. For different age groups, the elderly had a much higher level of IOp in inpatient expenditures than younger individuals, accounting for as much as 53.2% of total inequality. The regression results further showed that, older individuals with higher incomes or living in a community with a hospital had larger inpatient expenditures, while the effects were absent in younger individuals. These findings suggest that for older individuals, income and the availability of hospitals in the community were important sources of inequity in inpatient expenditures. According to our data, the average expenditure by the elderly on inpatient care was 18.3% higher than the average expenditure of younger persons, but only 6.5% more in the case of outpatient services. These figures indicated that older individuals had a higher level of demand for medical services than younger individuals, especially for inpatient services. It is necessary for policy makers to pay more attention to ensuring fairness in inpatient expenditures for the elderly.

### Limitations

There were a number of potential limitations to this paper. First, there were data limitations as the study period was confined to 5 years, 2011–2015. This period made it difficult to discern comprehensively time trends in inequality. Notwithstanding the length of the study period, the CHARLS provides information on both outpatient and inpatient expenditures, which allows us to achieve the main study objective, i.e. to measure IOp in expenditures for different categories of medical expenditures rather than just for total expenditures. Second, we were not able to include all the circumstance variables due to survey data limitations, the IOp value we measured was in fact the lower bound of the real IOp [51]. But given the CHARLS covered 28 provinces out of 31 provinces in China, it allowed for the assessment of regional differences in supply-side factors. Third, inpatient expenditures may be associated with more detailed need variables, such as the prevalence of disease, than just those included in this study. The absence of these variables from the CHARLS may explain why the need variables included in our model were not statistically significant determinants of inpatient expenditures. Fourth, we used having supplementary medical insurance as a proxy for a preference for the use of medical care, but it may also reflect the underlying need for care due to potential adverse selection effects. As our study focused on inequality due to circumstance, it did not matter whether supplementary insurance was a proxy for preference or need, as it did not affect our IOp measure.

## Conclusions

China has made remarkable progress in improving equity in both outpatient and inpatient expenditures for those over 45 years of age over the study period, 2011 to 2015. However, significant inequality of opportunity arising from social background and medical supply still exists and represents a challenge for all levels of government. This study provides information on which to base policies designed to reduce inequity in healthcare expenditures. Although the Chinese government launched the New Co-operative Medical System (NCMS) that provides financial assistance to vulnerable groups in rural regions, the benefit level warrants improvement so that it is in line with the other basic medical insurance schemes. More subsidies need to be used to support the NCMS. It is important to address the observed wide regional variations in medical expenditures. Options include reallocations of medical personal and resources to under-served regions and enhanced financial support for medically undeveloped regions. Additionally, we found a negative correlation between the availability of primary clinics and outpatient expenditures, but a positive correlation between hospital availability and outpatient expenditures. This finding suggests that, increasing access to quality primary community clinics may improve equity in the use of outpatient care, as it’s the associated lower medical costs at such clinics represent a pro-poor policy.

We also found that there existed greater inequity in inpatient expenditures when compared to outpatient expenditures. This finding suggests policies designed to protect vulnerable populations need to pay more attention to the financing and design of inpatient services. Finally, it is worth noting that the elderly, as a group with high demands for inpatient services, faced more serious inequity in inpatient expenditures. This finding highlights the importance of tailored policies to protect the elderly who are in poor living conditions, especially in the context of aging.

## Abbreviations

IOp: Inequality of opportunity
SES: Socioeconomic status
UEBMI: Urban Employee Basic Medical Insurance
URBMI: Urban Resident Basic Medical Insurance
NCMS: New Co-operative Medical System
CHARLS: China Health and Retirement Longitudinal Study
HRS: Health and Retirement Study
ELSA: English Longitudinal Study of Aging
SHARE: Survey of Health, Aging and Retirement in Europe
PPS: Probabilities proportional to size; SAH: Self-assessed health
